# Isobavachalcone Activates Antitumor Immunity on Orthotopic Pancreatic Cancer Model: A Screening and Validation

**DOI:** 10.3389/fphar.2022.919035

**Published:** 2022-08-25

**Authors:** Xuanming Liu, Hongbo Zhang, Jianlin Cao, Yuzhen Zhuo, Jiahui Jin, Qiaoying Gao, Xiangfei Yuan, Lei Yang, Dihua Li, Yan Wang

**Affiliations:** ^1^ College of Pharmaceutical Engineering of Traditional Chinese Medicine, Tianjin University of Traditional Chinese Medicine, Tianjin, China; ^2^ Tianjin Key Laboratory of Acute Abdomen Disease Associated Organ Injury and ITCWM Repair, Tianjin Nankai Hospital, Tianjin, China; ^3^ Department of Gynaecology and Obstetrics, Shanxi Provincial People’s Hospital, Shanxi, China; ^4^ State Key Laboratory of Component-based Chinese Medicine, Tianjin, China

**Keywords:** isobavachalcone, pancreatic cancer, tumor microenvironment, network pharmacology, bioinformatics, molecular docking

## Abstract

Pancreatic cancer is accompanied by poor prognosis and accounts for a significant number of deaths every year. Since *Psoralea corylifolia* L. (PCL) possesses a broad spectrum of bioactivities, it is commonly used in traditional Chinese medicine. The study explored potential antitumor agents of PCL and underlying mechanisms *in vitro* and *vivo*. Based on network pharmacology, bioinformatics, and molecular docking, we considered isobavachalcone (IBC) as a valuable compound. The activity and potential mechanisms of IBC were investigated by RT-qPCR, immunohistochemistry, immunofluorescence, and flow cytometry. It was confirmed that IBC could inhibit Panc 02 cell proliferation and induce apoptosis *via* increasing the production of reactive oxygen species. IBC could attenuate the weight of solid tumors, increase CD8^+^ T cells, and reduce M2 macrophages in the tumor tissue and spleen. Another promising finding was that IBC alleviated the proportion of myeloid-derived suppressor cells (MDSCs) in the tumor tissue but had no change in the spleen. The study of pharmacological effects of IBC was carried out and suggested IBC restrained M2-like polarization of RAW 264.7 cells by inhibiting the expression of ARG1 and MRC1 and suppressed the expression of ARG1 and TGF-β in bone marrow-derived MDSC. In summary, this research screened IBC as an antineoplastic agent, which could attenuate the growth of pancreatic cancer *via* activating the immune activity and inducing cell apoptosis. It might be a reference for the antitumor ability of IBC and the treatment of the tumor microenvironment in pancreatic cancer.

## Introduction

Research on pancreatic cancer has a long tradition. It is a devastating malignant disease and accounts for at least 3,31,000 deaths every year (Ilic and Ilic, 2016). Convincing data from the previous study have demonstrated that the current therapies, including surgery, radiotherapy, and chemotherapy, only warrant no more than 9% 5-year survival and a 26-month median survival (Dreyer et al., 2017). There are growing appeals for exploring a new therapeutic strategy, such as immunotherapy and combined treatment, to increase the survival rates of patients on pancreatic cancer.

Over the past two decades, one of the most popular opinions about pancreatic cancer is considered a “non-immunogenic” tumor. Nevertheless, recent preclinical data supports that pancreatic cancer can adopt numerous mechanisms to escape immune, including the secretion of immunosuppressive chemokines and cytokines, recruiting of regulatory immune cells, and the expression of cell surface proteins (Martinez-Bosch et al., 2018; Karamitopoulou, 2019). In addition, there are several unfavorable immune subsets that characterize their potent immune suppressive activity in the tumor microenvironment, which involved in T regulatory cells, alternatively activated M2 macrophages, and myeloid-derived suppressor cells (MDSC) by preventing the antitumor activity of effectors CD4^+^ T cells and CD8^+^ T cells (Gun et al., 2019).

**FIGURE 1:**
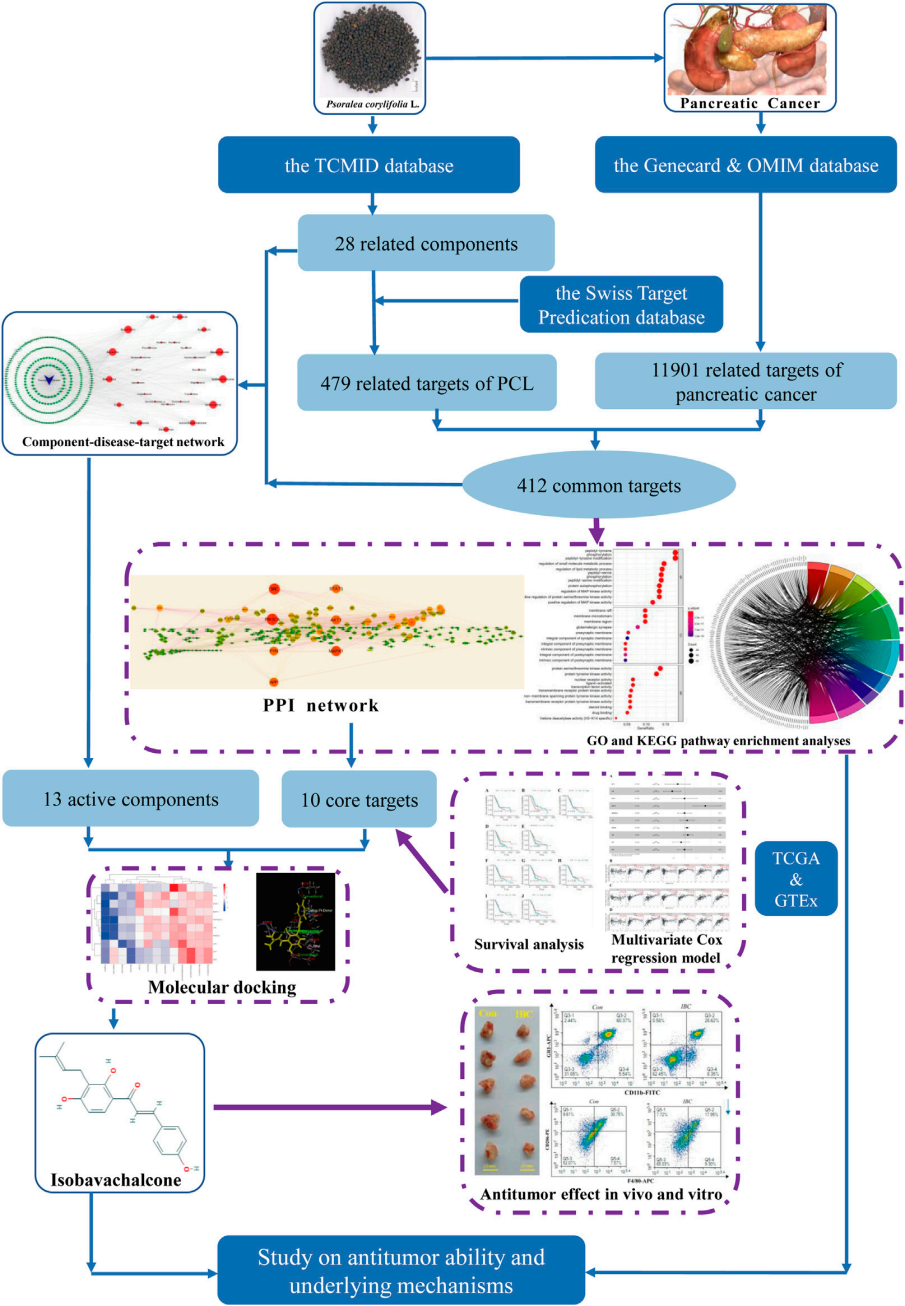
Research procedures applied in the study searching for an antitumor agent and their potential mechanisms.

*Psoralea corylifolia* L. (PCL) possesses excellent efficacy in the treatment of psoriasis and vitiligo and is commonly used in traditional Chinese medicine (TCM) (Yan et al., 2010; Zhou et al., 2019). Since TCM possesses multi-component and multi-target characteristics, PCL has a broad spectrum of bioactivities, including cytotoxic, antibacterial, antifungal, antitumor, and antioxidant (Alam et al., 2018). Isobavachalcone (IBC) was first extracted from PCL in 1968 and defined as a prenylated chalcone (Kuete and Sandjo, 2012). At present, theoretical research has revealed that IBC can inhibit colorectal cancer proliferation, induce apoptosis of prostate cancer PC-3 cells, and promote pro-caspase-3 and pro-caspase-9 in neuroblastoma (Li et al., 2018; Li et al., 2019; Nishimura et al., 2007). It selectively induces leukemic cell death with massive cytoplasmic vacuolation but no impairment to the normal peripheral blood cells (Yang et al., 2019). The aforementioned findings support the hypothesis that IBC is a promising antitumor agent. As far as we know, no previous research has investigated the antitumor ability of IBC and the underlying molecular mechanisms against pancreatic cancer. Our research idea is shown in Figure 1.

## Materials and Methods

### Data Source and Preprocessing

For this study, PCL components were derived from the Traditional Chinese Medicine Integrated Database (TCMID, http://www.megabionet.org/tcmid/) and duplicates were removed according to CAS Registry Number and PubChem CID (Huang et al., 2018). We collected the SMILES format of PCL components from the PubChem (https://pubchem.ncbi.nlm.nih.gov/) database. The targets for the therapeutic effects of PCL were obtained from the Swiss Target Prediction (http://www.swisstargetprediction.ch/), and the cutoff was set as probability >0. The GeneCards (https://www.genecards.org/) database and the OMIM (https://www.omim.org/) database provided relative pancreatic cancer targets. “Pancreatic Cancer” and “*Homo sapiens*” were programmed when searching for relative targets, and then targets were standardized into gene symbols. We used the intersection of two parts of targets and visualized it as a Venn plot.

### Construction of the Compound–Disease–Target Network

The overlapping targets were integrated into Cytoscape V3.8.2, which served as a platform for constructing the compound-disease-target network and visualizing their relationship (Alam et al., 2018). Topological arguments of the network were calculated by undirected graph analysis of the network, such as degree, closeness centrality, and betweenness centrality. We screened active compounds with the median of the abovementioned arguments, which can be used as an optimal threshold to assess node importance.

### Analysis of the Protein–Protein Interaction and Expression Profile of Core Targets

This study applied a protein-protein interaction (PPI) network to identify the interaction between PCL component targets and pancreatic cancer-related targets. The overlapping targets were imported into Cytoscape StringApp V1.6.0 based on the STRING database (https://string-db.org/) (Alam et al., 2018; Szklarczyk et al., 2021), and two crucial options were programmed to be “*H. sapiens*” and “highest confidence (0.900).” We sorted all targets by the aforementioned topological arguments, identified the top 10 core targets, and constructed the expression profile of core targets between tumor and normal tissues on the basis of The Cancer Genome Atlas (TCGA) database and the Genotype-Tissue Expression (GTEx) database.

### Gene Ontology and Kyoto Encyclopedia of Genes and Genomes Enrichment Analyses

We applied Gene Ontology (GO) functional enrichment and Kyoto Encyclopedia of Genes and Genomes (KEGG) pathway enrichment analyses to search for biological function, cell location, and potential mechanisms by the R package: clusterProfiler (Yu et al., 2012). The statistical significance threshold was set at *p* value <0.01 and *q* value <0.05. The outcomes of GO and KEGG pathway enrichments were presented by the R package: GOplot (Walter et al., 2015).

### Prognostic Value of Core Targets for the Evaluation of Pancreatic Cancer

We used UCSC Xena to obtain gene expression and survival data of pancreatic cancer patients at TCGA and categorized the samples into high- (50%) and low- (50%) cohorts with the median value of expression (Goldman et al., 2020; Wang et al., 2021). The multivariate Cox proportional hazards model was also performed to determine the independent prognostic value of core targets. *p* < 0.05 was considered a statistically significant difference. The correlation between core targets and immune cells of infiltration was calculated based on the TIMER (https://cistrome.shinyapps.io/timer/) database (Li et al., 2017).

### Molecular Docking Strategy

The Discovery Studio 4.5 client is a ligand-receptor docking software generally used for estimating their complementarity by the scoring function. In the PDB database, the proper structures of core targets were searched and downloaded, which included the pleckstrin homology domain from human Akt (AKT1, PDB ID: 1H10), human Hsp90-alpha with 9-Butyl-8-(3,4,5-trimethoxy-benzyl)-9H-purin-6-ylamine (HSP90AA1, PDB ID: 1UY6), the crystal structure of the Fyn kinase domain complexed with staurosporine (FYN, PDB ID: 2DQ7), SH3 domain of human Lck (LCK, PDB ID: 2IIM), Lyn kinase domain (LYN, PDB ID: 3A4O), the crystal structure of human ERK2 complexed with a MAPK docking peptide (MAPK1, PDB ID: 4H3P), human Src A403T mutant bound to kinase inhibitor bosutinib (SRC, PDB ID: 4MXX), the E1-domain of the amyloid precursor protein (APP, PDB ID: 4PWQ), Stat3 Core in complex with compound SI109 (STAT3, PDB ID: 6NUQ), and the compound bound to the PI3Ka catalytic subunit p110alpha (PIK3CA, PDB ID: 6OAC). The SDF format files of active compounds were obtained from the PubChem database. We applied the CHARMm force field on the structures of ligands and minimized them to the closest local minimum. Subsequently, we defined the active site based on co-crystallized ligands, removed water molecules, added hydrogen atoms to macromolecules, and docked the active compounds into receptors in the pattern of semi-flexible docking. AutoDock Vina 1.1.2 is a widely accepted open-source program for molecular docking and virtual screening, which attempts to predict noncovalent binding between receptors and ligands. The pretreatment of macromolecules and small molecules and the specification of “search space” were similar to those mentioned in Discovery Studio. The compound that had the best conformation with receptors was considered a valuable candidate and further studied *in vivo*.

### Cell Lines and Cell Culture

The murine pancreatic cancer cell line Panc 02 and the mouse macrophage cell line RAW 264.7 cells were obtained from the American Type Culture Collection and employed for the experiment. The cells were incubated with Gibco Dulbecco’s modified Eagle’s medium with 100 μg/ml streptomycin and 100 U/ml penicillin and 10% fetal bovine serum (Biological Industries, Israel) in an incubator at 37°C and 5% CO_2_.

### Cell Proliferation Assay

According to the protocol, cell proliferation was monitored by the CCK-8 assay (Dojindo Laboratories, Kumamoto, Japan). Panc 02 cells were seeded in a 96-well plate and treated with different concentrations of IBC. After incubation for 24 h, the 10 μl of CCK-8 reagent was added and incubated for another 2 h at 37°C. Finally, the complex resolution was detected by a microplate at 450 nm.

### Apoptosis and ROS Assay

Administrated with IBC (10 μM, 20 μM) for 24 h, Panc 02 cells were collected and stained with Annexin V and propidium iodide (PI) according to the manufacturer’s instructions (Tianjin Sungene Biotech, China). Meanwhile, Panc 02 cells were stained with DCFH-DA to detect the level of ROS generation. Both were identified by flow cytometry (ACEA, United States).

### Orthotopic Model of Pancreatic Cancer

All animal experiments were reviewed and approved by the Medicine Ethical Committee of Tianjin Nankai Hospital (Approval No. NKYY-DWLL-2020-102). Orthotopic models of pancreatic cancer were established as described previously (Xi et al., 2020). Briefly, Panc 02 cells (5 × 10^5^), resuspended in 50 μl PBS, were injected into the pancreas of female 6- to 8-week-old C57BL/6 mice (the Experimental Animal Center of Military Medical Sciences, Beijing, China) by insulin syringe. On the third day after the model was established, IBC (20 mg/kg/day) was administered intraperitoneally for 10 days.

### Flow Cytometry

The tumor tissue was cut up into pieces and digested with enzymes for 30 min at 37°C, including 0.05 mg/ml of type-IV collagenase (Sigma, United States), DNase I (Sigma, United States), and hyaluronidase (Sigma, United States). The tumor tissue was ground into single cells through a 70 μm strainer. Tumor mononuclear cells were isolated by density gradient centrifugation, and the density gradient solution was 40% percoll and 80% percoll (GE, United States). The spleen tissue was ground into single cells through a 40 μm strainer (FALCON, United States). The further red blood cell lysate was used to remove blood cells. Tumor mononuclear cells and spleen cells were stained with the following antibodies: CD11b-FITC, Ly6G-PerCP, Ly6c-PE, CD4-APC, CD8-PE, NK1.1-PE, F4/80-APC, Gr-1-PE, and CD206-PE. Natural killer cells (NK, CD3^−^NK1.1^+^), MDSC cells (CD11b^+^Gr-1^+^), monocytic myeloid-derived suppressor cells (M-MDSC, Ly6C^+^CD11b^+^), polymorphonuclear myeloid-derived suppressor cells (PMN-MDSC, Ly6G^+^CD11b^+^), CD4^+^ T cells (CD3^+^CD4^+^), CD8^+^ T cells (CD3^+^CD8^+^), and M2 macrophage cells (CD11b^+^F4/80^+^CD206^+^) were detected and analyzed by NovoCyte (ACEA, United States).

### RNA Extraction and Real-Time PCR

According to the manufacturer’s instructions, total RNA was extracted from RAW264.7 cells using Trizol reagent (Takara, Japan). The mRNA was reverse transcribed into cDNA using cDNA Synthesis SuperMix (TransGen Biotech, China), and real-time PCR was carried out using SYBR Green Master Mix (TransGen Biotech, China). Quantitative fluorescence analysis was performed using the ABI 7500 Real-time PCR System (Thermo Scientific, United States). GAPDH was used as an internal reference, and relative gene expression was calculated using the 2^−ΔΔCt^ method. The primer sequences used in this study were as follows: Arginase 1 (ARG1) (F) 5′-CTC​CAA​GCC​AAA​GTC​CTT​AGA​G-3′ and ® 5′-GGA​GCT​GTC​ATT​AGG​GAC​ATC​A-3′; Mannose Receptor C-Type 1 (MRC1) (F) 5′-CTC​TGT​TCA​GCT​ATT​GGA​CGC-3′ a®(R) 5′-CGG​AAT​TTC​TGG​GAT​TCA​GCT​TC-3′; TGF-β (F) 5′-GGA​CCG​CAA​CAA​CGC​CAT​CTA​T-3′ and ®-β (R) 5′- TTC​AGC​CAC​TGC​CGT​ACA​ACT​C-3′; GAPDH (F) 5′-ATG​GTG​AAG​GTC​GGT​GTG​AAC​G-3′ a®GAPDH (R) 5′- CGC​TCC​TGG​AAG​ATG​GTG​ATG​G-3′.

### Isolation of Bone Marrow Cells

The bone was from healthy C57BL/6 mice. We cut off two ends and flushed out the cells, utilizing Gibco Dulbecco’s modified Eagle’s medium with 100 μg/ml streptomycin and 100 U/ml penicillin. Bone marrow-derived MDSC were induced using 40 ng/ml granulocyte-macrophage colony-stimulating factor (GM-CSF, PeproTech, United States) and 40 ng/ml interleukin 6 (IL-6, PeproTech, United States). At the same time, these cells were treated with IBC (10 μM) for 4 days.

### Western Blot Analysis

Total protein was lysed and isolated from Panc 02 cells. After determining the protein concentrations using the BCA assay, the proteins were purified by SDS-PAGE and transferred to polyvinylidene difluoride (PVDF) membranes. The membranes were blocked for 2 h at room temperature with 5% skim milk and incubated with primary antibodies overnight at 4°C. The membranes were incubated with HRP-bound secondary antibodies at room temperature for 2 h, and the protein bands were observed with ELC luminescent solution. The primary antibodies used are as follows: Bax, Bcl-2, and GADPH (Cell Signaling Technology, United States).

### Ki67 Staining

The tumor tissues were fixed with 4% paraformaldehyde, embedded in paraffin, and cut into 4-μm sections. Then the sections were incubated with primary antibodies for the proliferation marker protein Ki67 (anti-Ki67, Cell Signaling Technology, Boston, United States). Subsequently, the section was incubated with horseradish peroxidase-conjugated goat anti-rabbit immunoglobulin G (ZSJQB Co., Ltd., Beijing). Following incubation, hematoxylin was utilized to counterstain, and the tissues were observed under the inverted fluorescence microscope.

### Terminal Deoxynucleotidyl Transferase-Mediated dUTP Nick End Labeling Staining

The tumor tissues were fixed with 4% paraformaldehyde, embedded in paraffin, and cut into 4-μm sections. Then the sections were incubated with Terminal deoxynucleotidyl transferase dUTP nick end labeling (TUNEL) according to the manufacturer’s instructions (Yeasen Biotechnology, Shanghai). The nucleus was stained with DIPA for 5 min at room temperature without light, and the sections were observed under the inverted fluorescence microscope.

### Statistical Analysis

Statistical analysis was performed using R (version 4.03) and Rstudio software. Student’s *t*-test and one-way ANOVA were used to test the significance of differences *in vitro* and *vivo*. *p* < 0.05 was considered a gold standard of significant difference in this research.

## Results

### Data Source and Preprocessing

**FIGURE 2:**
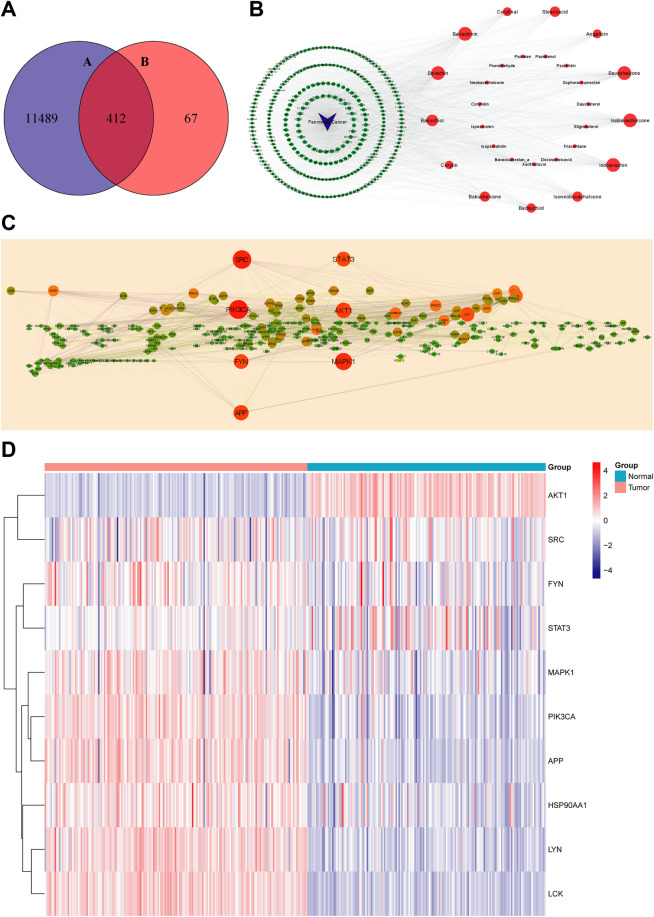
Virtual screening for core targets of PCL against pancreatic cancer. **(A)** Overlapping targets of PCL and pancreatic cancer: 11,901 targets of pancreatic cancer and 479 targets of PCL components. **(B)** Compound–disease–target network (red nodes represent PCL components, green nodes represent targets of pancreatic cancer, and blue nodes represent pancreatic cancer. The larger the node, the more important is the node in the network). **(C)** PPI network of PCL in the treatment of pancreatic cancer (the larger and redder node, the more critical is the node in the network). **(D)** Expression profile of the 10 core targets between the normal and tumor tissues (blue: low expression level; red: high expression level).

In this research, 36 compounds were initially obtained from TCMID. After removing two duplications and six compounds without any predicted targets, the integrated information of structures and predicted targets is shown in Supplementary Table S1. Absorbed prototype compounds are the most common pattern of TCM worked *in vivo* and therefore predicted to construct the network (Tong et al., 2021). In total, 479 targets of PCL were obtained by the SwissTargetPrediction database. Subsequently, “Pancreatic Cancer” was submitted to the OMIM and GeneCard databases for searching relative targets, and 11,489 targets were acquired. Then, 412 overlapping targets between compounds and pancreatic cancer were considered as potential treatment targets and further analyzed (Figure 2A).

### Construction of the Compound–Disease–Target Network

The network consisted of 441 nodes (28 compound nodes, 412 overlapping target nodes, and one disease node) and 2,296 edges (Figure 2B). Larger nodes represent higher correlation and importance. The hub nodes were characterized by degree ≥ 35, betweenness centrality ≥ 0.002, and closeness centrality ≥ 0.36. In total, it contained bavachalcone, bavachin, bavachinin, isobavachalcone, isobavachin, bakuchiol, stearic acid, isoneobavachalcone, bakuchalcone, backuchiol, angelicin, corylinal, and corylin (Supplementary Table S2). These compounds might be the active compounds of PCL in the treatment of pancreatic cancer.

### Analysis of the PPI Network and Expression Profile of Core Targets

We analyzed 412 overlapping targets and constructed the PPI network (Figure 2C). The larger and redder the node, the more vital interaction is. There were 299 nodes and 1,956 edges calculated and ranked by topological arguments. The top 10 targets were considered core targets, including PIK3CA, SRC, MAPK1, AKT1, APP, STAT3, FYN, LCK, LYN, and HSP90AA1 (Supplementary Table S2). Furthermore, the expression profiles of 10 core targets in 182 tumor tissues and 165 normal tissues are presented as a heatmap in Figure 2D.

### Gene Ontology and Kyoto Encyclopedia of Genes and Genomes Enrichment Analyses

**FIGURE 3:**
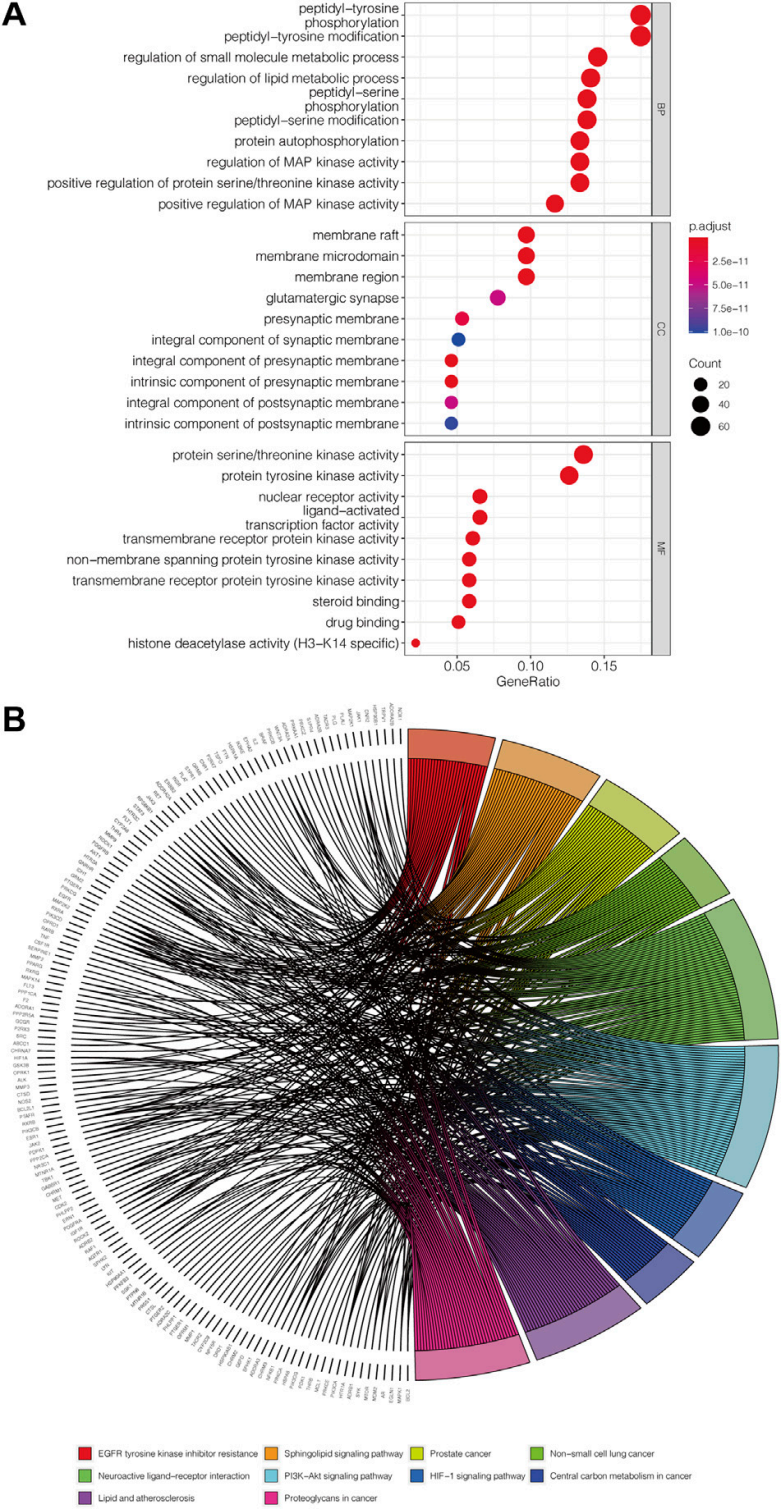
Potential approach of PCL against pancreatic cancer. **(A)** Secondary classification dot plot of GO functional enrichment analysis including biological process (BP), cellular components (CC), and molecular function (MF). The abscissa shows the GO term, and the ordinate shows the numbers of genes enriched in the GO term. **(B)** Chord plot of the relationship of the selected gene and their corresponding KEGG terms. The colors display different terms, and the angle of the chord represents the number of genes enriched in the KEGG pathway.

The 412 overlapping targets were imported for GO functional enrichment and KEGG pathway enrichment analysis. The top 30 GO terms are obtained and presented in Figure 3A, consisting of 10 biological processes (BP), 10 cellular components (CC), and 10 molecular functions (MF) terms. The targets of BP terms mainly dealt with peptidyl-tyrosine phosphorylation, regulation of small molecule metabolic processes, regulation of lipid metabolic processes, peptidyl-tyrosine modification, protein autophosphorylation, and regulation of MAP kinase activity. The CC terms mainly included membrane raft, membrane microdomain, and membrane region. The results of MF terms showed that the targets were mainly connected with protein serine/threonine kinase activity, protein tyrosine kinase activity, nuclear receptor activity, ligand-activated transcription factor activity, and transmembrane receptor protein kinase activity. On the other hand, we obtained 143 KEGG pathway items and selected the top 10 pathways to visualize (Figure 3B). The main pathways included the PI3K-Akt signaling pathway, EGFR tyrosine kinase inhibitor resistance, Neuroactive ligand-receptor interaction, Sphingolipid signaling pathway, and lipid and atherosclerosis.

### Prognostic Value of Core Targets for the Evaluation of Pancreatic Cancer

**FIGURE 4:**
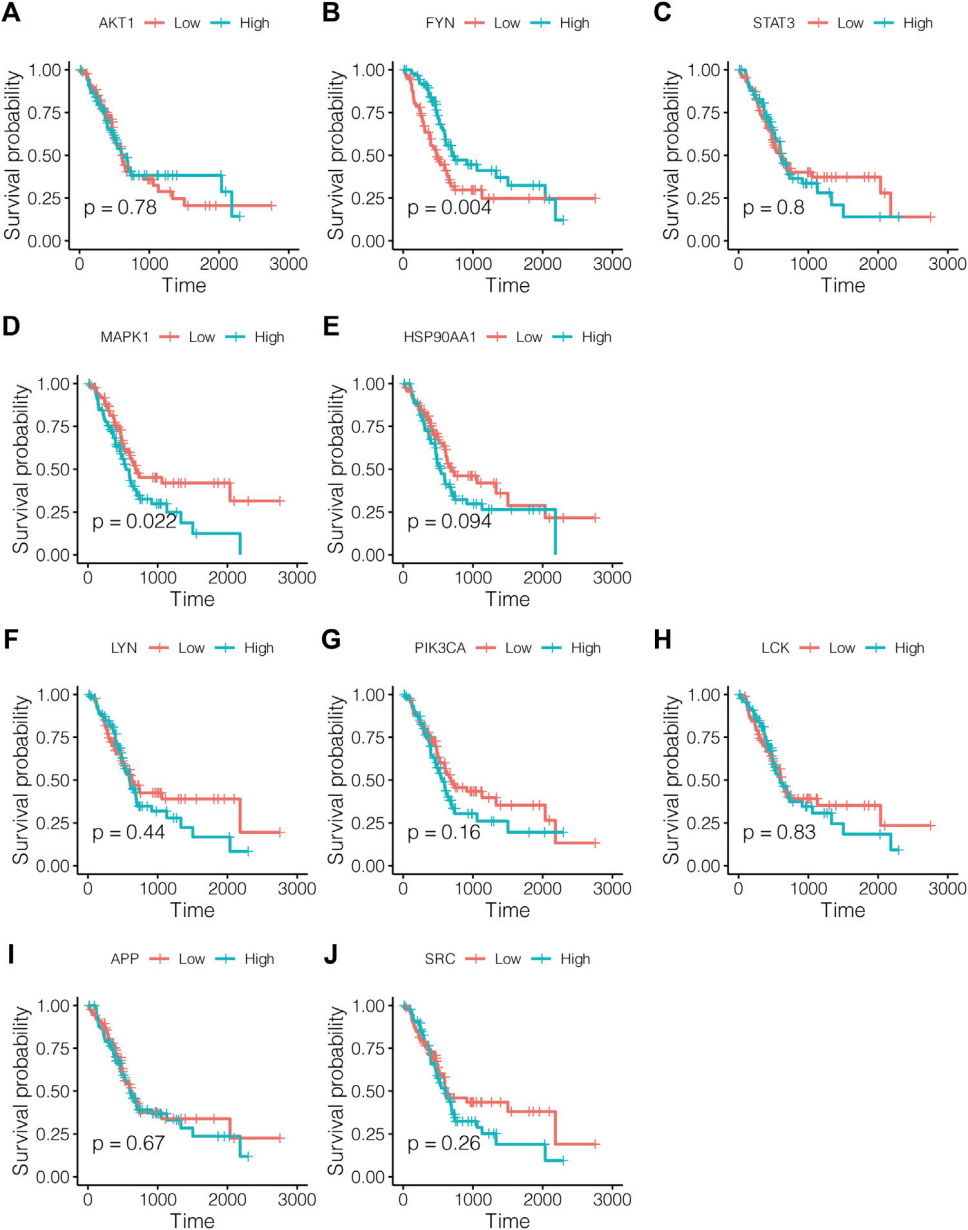
Survival analysis for study effects of 10 core targets in pancreatic cancer patients. Red lines represent sample groups with low gene expression, while blue lines represent sample groups with high gene expression. **(A)** AKT1, **(B)** FYN, **(C)** STAT3, **(D)** MAPK1, **(E)** HSP90AA1, **(F)** LYN, **(G)** PIK3CA, **(H)** LCK, **(I)** APP, and **(J)** SRC.

**FIGURE 5:**
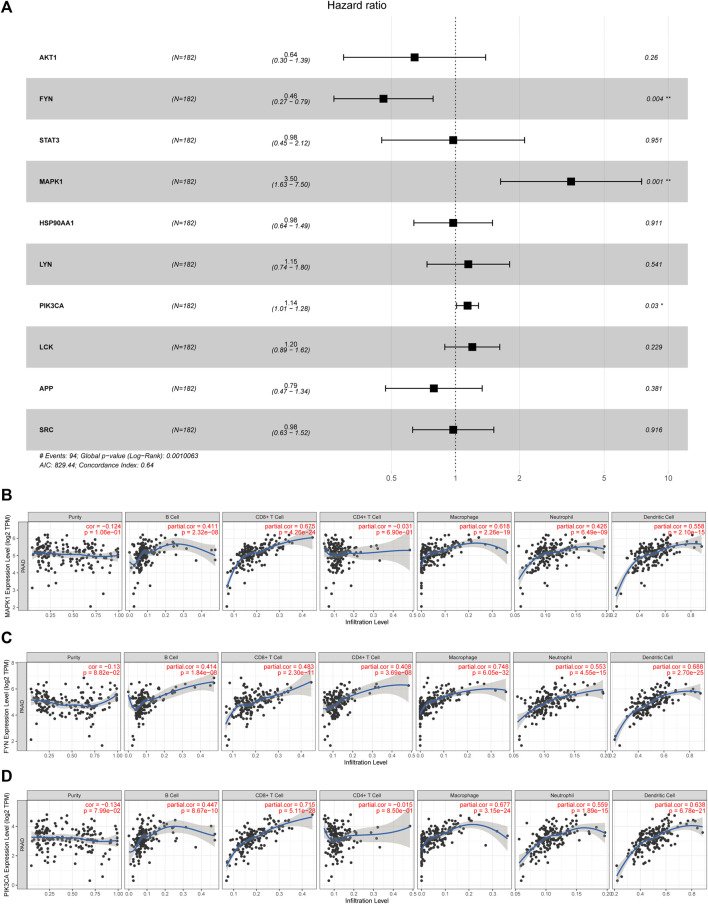
Identification of core targets with a significant prognostic value in pancreatic cancer. **(A)** Forest plot showed HR (95% CI) and *p*-value of core targets by multivariate Cox proportional hazards model; *p < 0.05 and **p < 0.001. **(B–D)** Correlations of the target expression level and immune cell infiltration (purity, B cells, CD8^+^ cells, CD4^+^ cells, macrophages, neutrophils, and dendritic cells).

As shown in Figure 4, lowly expressed FYN (*p* = 0.004) and highly expressed MAPK1 (*p* = 0.022) are associated with poor prognosis, and the correlations of the other core targets and overall survival time are not tested for statistical significance (*p* < 0.05). Subsequently, the multivariate Cox regression model also revealed that FYN, MAPK1, and PIK3CA possessed independent prognostic value (Figure 5A). The further novel funding of the TIMER database was that the expression level of FYN, MAPK, and PIK3CA correlate with immune infiltrates of B cell, CD8^+^ cell, macrophage, neutrophil, and dendritic cell in pancreatic cancer patients (Figures 5B–D). It suggested that ten targets achieved by virtual screen were reliable, and the antitumor activity of PCL probably depended on regulating the immune system in the tumor microenvironment.

### Molecular Docking of Active Compounds and Core Targets

**FIGURE 6:**
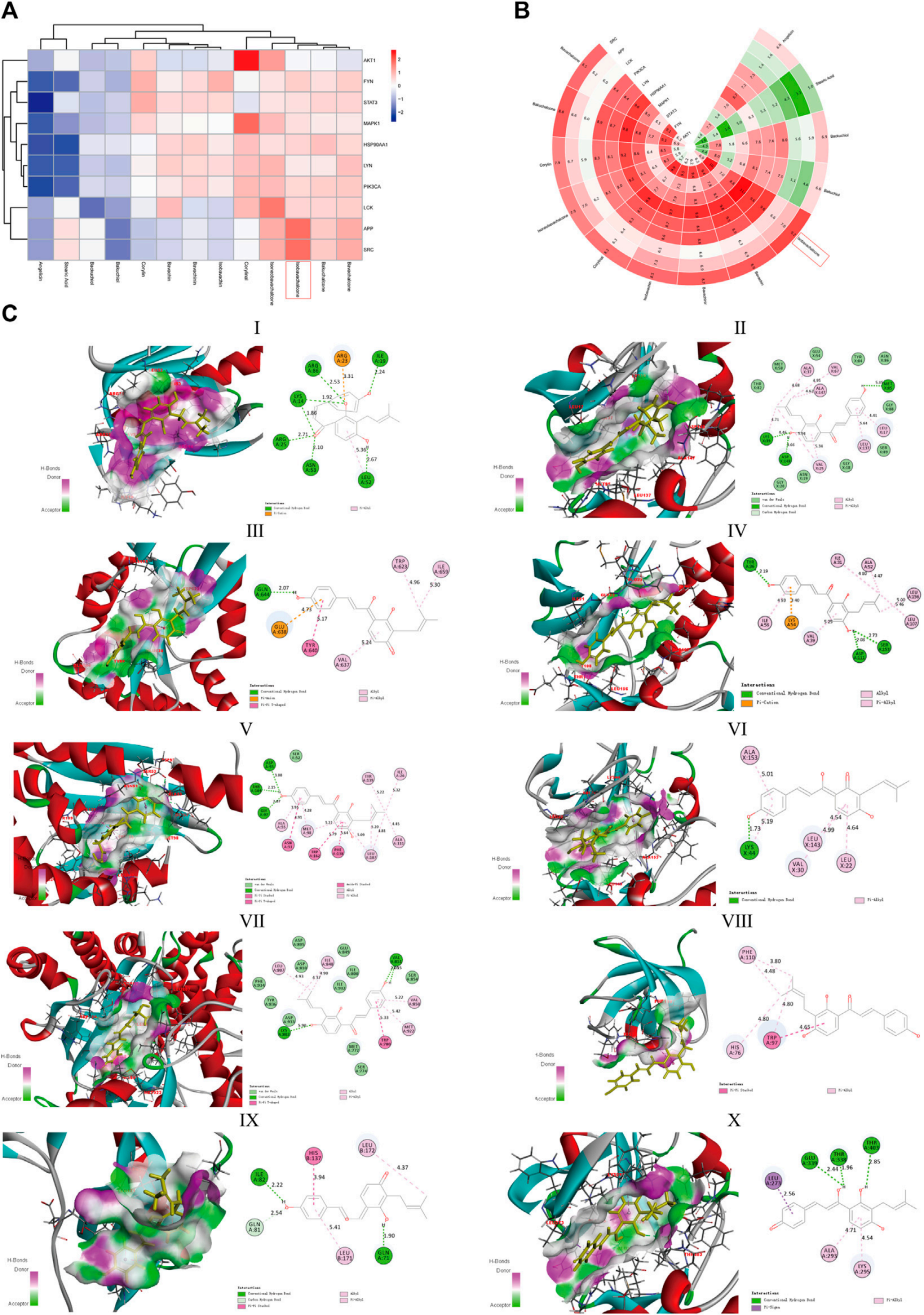
Molecular docking of components and targets. **(A)** Heatmap of the absolute value of the scores from Discovery Studio with Z-score normalized. **(B)** Polar heatmap of the absolute value of the scores from AutoDock Vina with Z-score normalized. The red node refers to a stable docking conformation, while blue or green representing poor interactions, which is suitable for **(A,B)**. **(C)** Docked conformation between IBC and 10 core targets. (Ⅰ) AKT1, (Ⅱ) HSP90AA1, (Ⅲ) FYN, (Ⅳ) LCK, (Ⅴ) LYN, (Ⅵ) MAPK1, (Ⅶ) SRC, (Ⅷ) APP, (Ⅸ) STAT3, and (Ⅹ) PIK3CA.

Following the aforementioned screening, we acquired 13 active compounds and 10 core targets in the compound-disease-target network and applied molecular docking between ligands and receptors. The “CDOCKER_INTERACTION_ENERGY” represents the level of interaction between the compound and the receptor. The lower the energy, the better the interaction is. Vina uses the scoring function “Affinity” to approximate the standard chemical potentials of the system. Both the scores of Discovery Studio and AutoDock Vina were normalized with Z-score in Figure 6A and Figure 6B, respectively. The best representative conformations are presented in Figure 6C. In which IBC possessed the better docking scores and the lower root-mean-square deviation in two programs simultaneously, hence we considered IBC a valuable candidate. Moreover, we paid close attention to the conformation of FYN, MAPK1, and PIK3CA. FYN is a non-receptor Tyr kinase and belongs to the Src family of kinases (Demuro et al., 2021). The 2D diagram showed that IBC formed hydrogen bonds with the hinge residues of FYN (Met85) and reached into the hydrophobic pocket, which had hydrophobic interaction with lots of residues: Leu17, Val25, Ala37, Lys39, Val67, Leu137, and Ala 147 (Zhang et al., 2021). It was in line with the Src family kinase inhibitor. We described the docking result of FYN and showed these hydrogen bonds and hydrophobic interactions could facilitate IBC binding to FYN and might exert an inhibitory effect. MAPK1, an alias for extracellular signal-regulated kinase 2, is a member of the mitogen-activated protein kinase family. It has been shown that Asp111, Ser153, and Cys166 in the ATP-binding site of ERK2 were the important residues and contributed to being a selective inhibitor of ERK2 (Aly et al., 2018). Our findings on molecular docking hint that IBC might inhibit the activity of MAPK1 via forming hydrogen bonds with Asp111 and Ser153. PIK3CA encodes p110α, which is the catalytic subunit of phosphatidylinositol 3-kinase *α* (PI3Kα) and is somatically mutated high frequently (Miller et al., 2019). In our study, the docking in PI3Kα highlighted that IBC formed hydrogen bonds with Lys802 and Pi-Pi T-shaped interaction with Trp780 and possessed van der Waals with Asp805 and Asp810, which are important residues involved in the ATP pocket (Han and Zhang, 2010).

### Isobavachalcone Inhibited Cell Proliferation and Induced Cell Apoptosis of Panc 02 Cells

**FIGURE 7:**
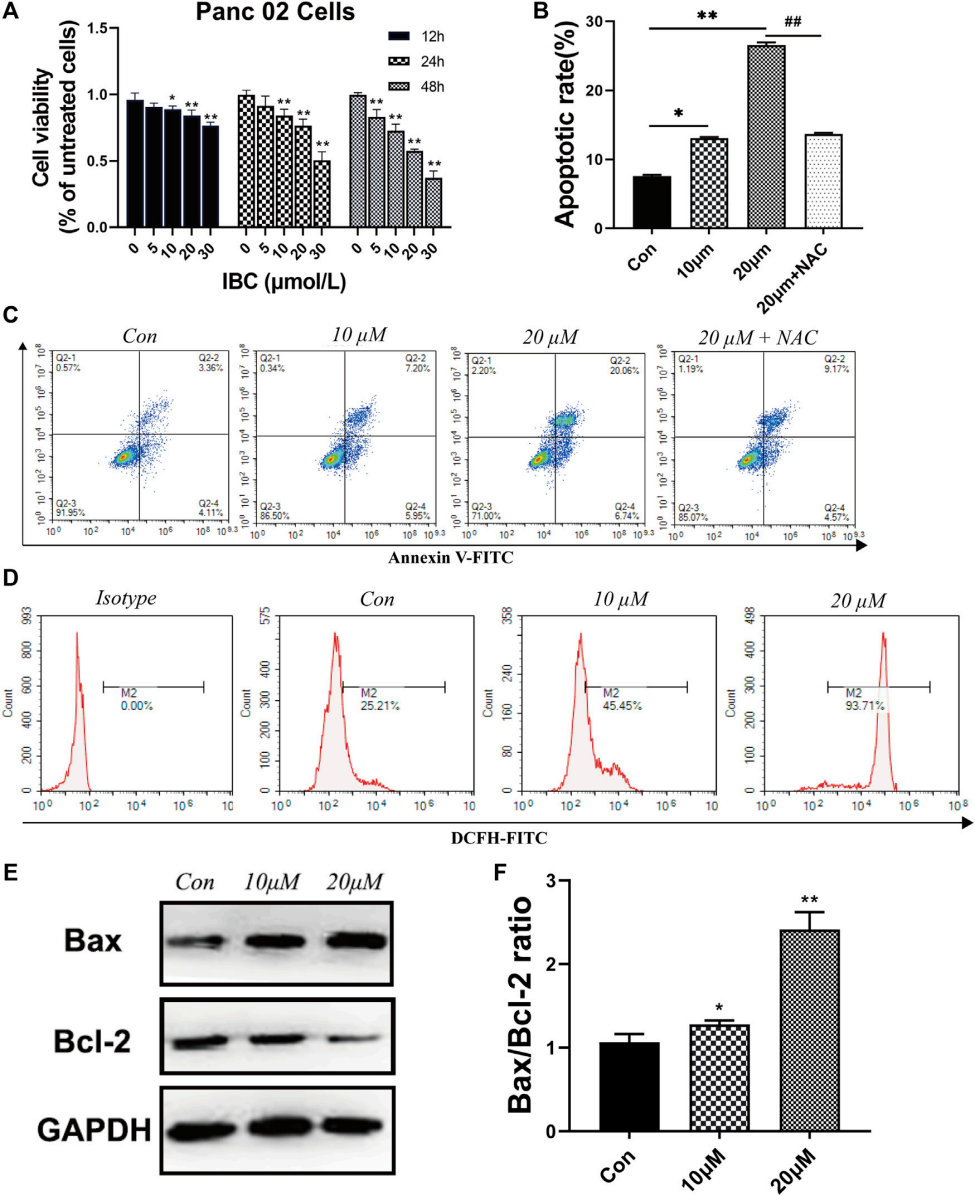
IBC exerted the effects of inhibiting proliferation and promoting apoptosis. **(A)** CCK8 assays of Panc 02 cells after treatment with IBC (1 μM, 5 μM, 10 μM, 15 Μm, 20 μM, and 30 μM) for 12, 24, and 48 h; *p < 0.05 and **p < 0.001, versus the control group. **(B‐C)** The static cell apoptosis rates were detected by flow cytometry (*N* = 3). The representative image is reported. The columns represent the mean ± SEM. Student’s t test was applied to analyze the data, *p < 0.05 **p < 0.001 versus the control. **(D)** Flow cytometry images and fluorescence intensity of ROS staining (*N* = 3). The representative image is reported. **(E,F)** Western blot results of Bax, Bcl‐2, and GAPDH after being treated with 10 μM and 20 μM. The columns represent the mean ± SEM, *p < 0.05 **p < 0.001, versus the control group.

The study explored the biological activities of IBC on pancreatic cancer and put IBC with different concentrations into the cellular model. The CCK-8 assay demonstrated that IBC significantly inhibited the proliferation abilities of Panc 02 cells at 24, 48, and 72 h versus the control group (Figure 7A). Then, flow cytometry indicated that IBC treatment with various concentrations for 24 h triggered cell apoptosis and ROS generation (Figures 7B–D). Since the role of ROS in the process of IBC-induced apoptosis remained uncovered, NAC, a ROS scavenger, was applied prior to IBC treatment, and it was shown that NAC pretreatment abrogated apoptosis produced by IBC in Panc 02 cells. In an apoptosis assay, we also found that IBC could decrease the expression of Bcl-2 and increase expression of Bax (Figures 7E,F). This suggested that IBC could inhibit the proliferation and induce apoptosis of pancreatic cancer cells by increasing the generation of ROS.

### Isobavachalcone Suppressed Orthotopic Pancreatic Cancer in Mice

**FIGURE 8:**
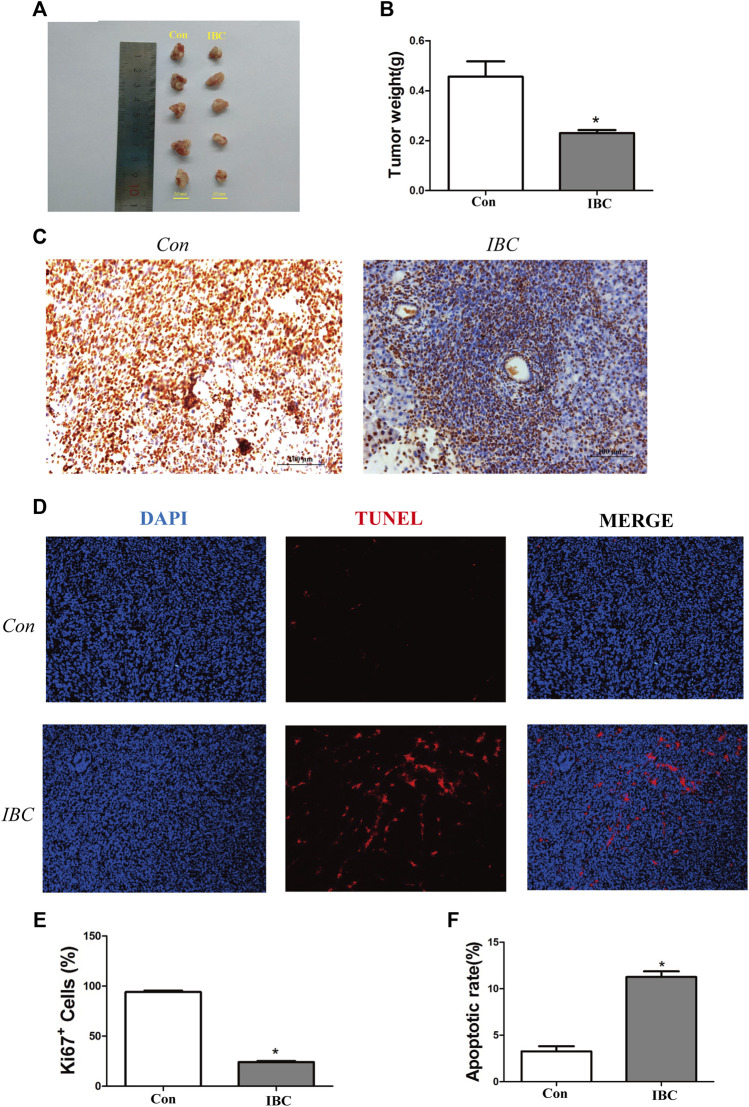
IBC slowed the growth of pancreatic cancer *in vivo*. **(A,B)** Tumor tissues were isolated from the orthotopic models (*N* = 5). The therapeutic mice with orthotopic pancreatic tumors were treated with IBC (20 mg/kg) for 10 days. The columns represent the mean ± SEM. Student’s *t*-test was applied to analyze the data; *p < 0.05 versus the control. **(C)** Expression of Ki67 in tissues was valued through immunohistochemistry (scale: 100 μm). **(D)** TUNEL staining showed the proportion of apoptotic cells in tissues (scale: 100 μm). **(E)** Quantitative analysis of the Ki67^+^ cells in tumor tissues, *p < 0.05 versus the control. **(F)** Quantitative analysis of the apoptosis rate in tumor tissues, *p < 0.05 versus the control.

The present study established the orthotopic pancreatic cancer model in mice and treated them with IBC for 10 days. The tumor weight decreased significantly after IBC treatment at 10 days versus the control group (Figures 8A,B), and the immunohistochemistry for Ki67 staining indicated that IBC suppressed cell proliferation (Figures 8C,E). In addition, the immunofluorescence images with TUNEL staining showed that the proportion of apoptotic cells was directly elevated by IBC *in vivo* (Figures 8D,F). From the short review mentioned earlier, key findings emerged that IBC could slow the growth of tumors by promoting apoptosis and reducing proliferation.

### Isobavachalcone Inhibited the Expansion of M2 Macrophages and MDSC in Mice

**FIGURE 9:**
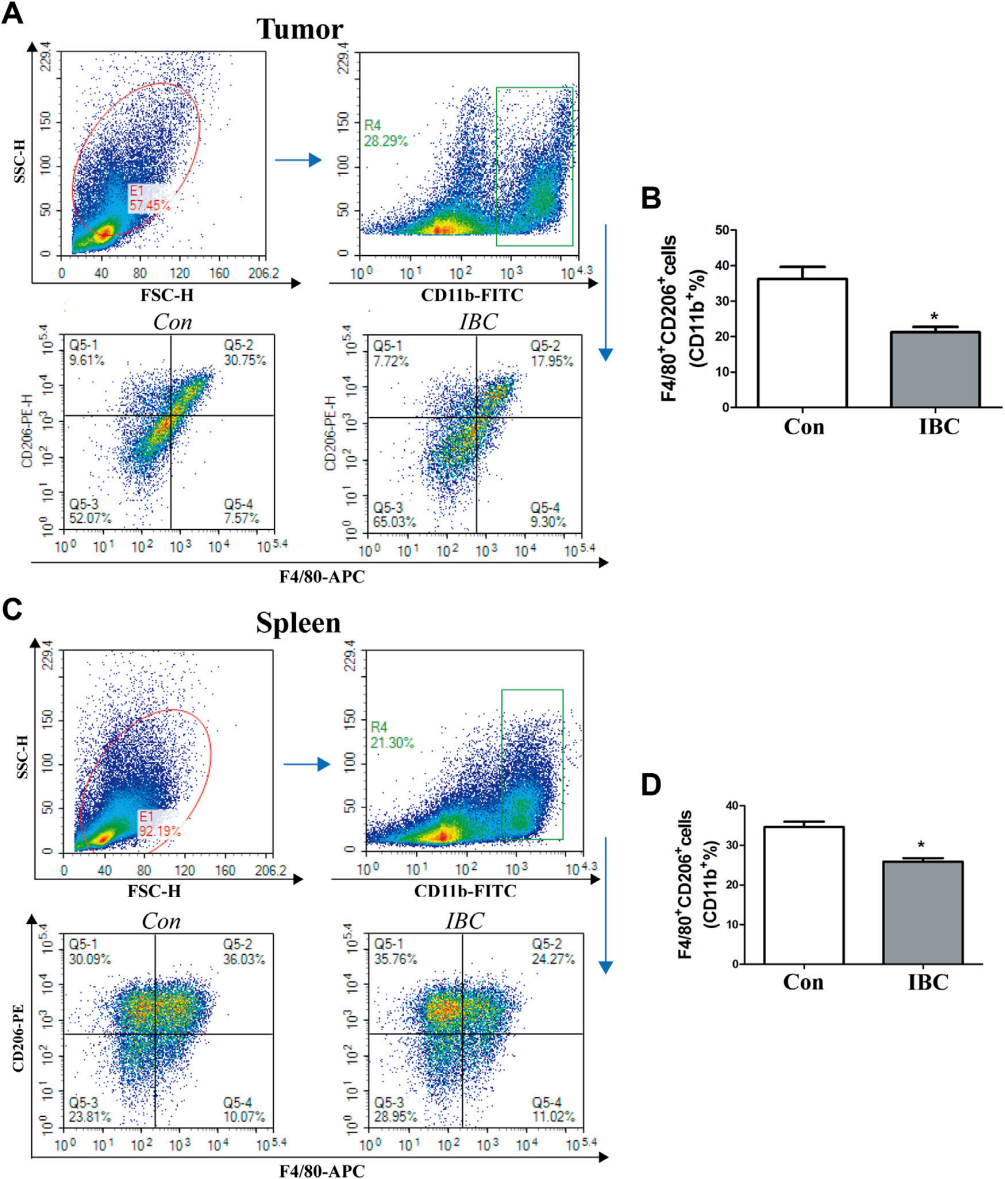
Effects of IBC on M2-like macrophage polarization. **(A,B)** M2 macrophages polarized in the tumor tissue (*N* = 5), *p < 0.05 versus the control. **(C,D)** M2 macrophages polarized in the spleen (*N* = 5), *p < 0.05 versus the control.

**FIGURE 10:**
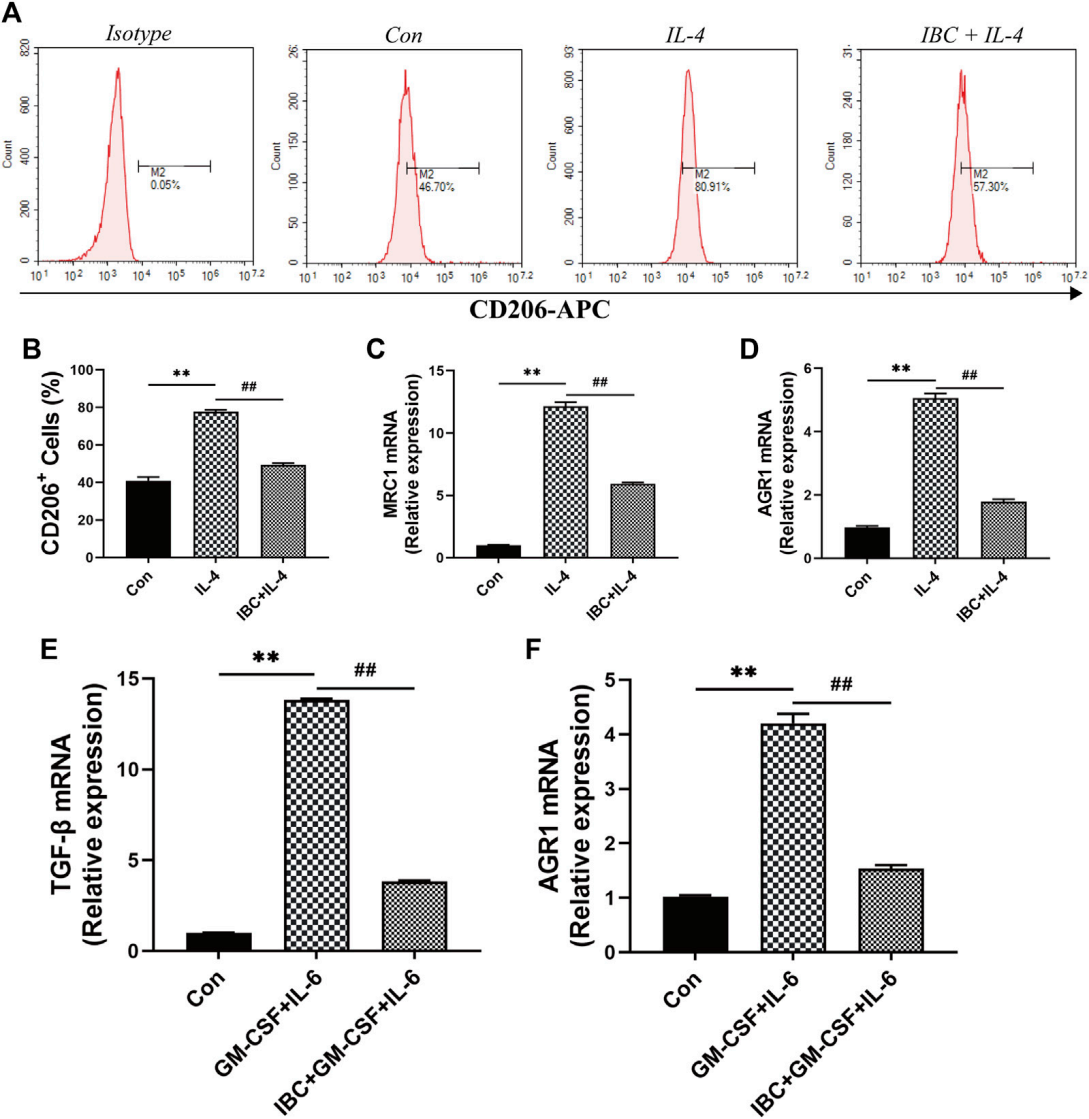
The mechanism of IBC reduced the proportion of M2 macrophages. **(A)** RAW 264.7 macrophages were stimulated with IL-4 (20 ng/mL) and then treated with IBC (10 μM) for 4h. **(B‐D)** Comparison of CD206+ cells in the IL-4 + IBC group and the IL-4-stimulated group alone, IBC reduced the mRNA expression of ARG1 and MRC1 in M2 macrophages, **p < 0.01 versus the control group and ##*p* < 0.01 versus the IL-4 group. **(E,F)** Comparison of MDSC in the GM-CSF + IL-6 group and the IBC + GM-CSF + IL-6 group, IBC reduced the mRNA expression of TGF-β and AGR1, **p < 0.01 versus the control group and ##*p* < 0.01 versus the GM-CSF + IL-6 group.

In the tumor microenvironment, the components such as M2 macrophages and MDSC infiltrated and promoted occurrence and metastasis of cancer cells (Lorenzo-Sanz and Muñoz, 2019). Our findings on the tumor tissues and spleen hint that IBC could significantly reduce the accumulation of M2 macrophages (Figures 9A–D). We explored the mechanism of polarization in the presence or absence of IBC (10 μM) in RAW 264.7 cells and found that IBC inhibited the cells into M2 polarization and reduced the expression of ARG1 and MRC1 (Figures 10A–D). Another promising finding was that IBC cut down the proportions of MDSC in tumor tissue but not in spleens (Supplementary Figure S1). Since MDSC mainly performed immunosuppressive effects by expressing ARG1 and TGF-β, we used GM-CSF and IL-6 to induce MDSC from the bone marrow and detect the transcription of ARG1 and TGF-β (Figures 10E,F). These data suggest that IBC could attenuate the expansion of M2 macrophages and MDSC in the tumor tissue by inhibiting the expression of ARG1, MRC1, and TGF-β.

### Isobavachalcone Promoted the Population of CD8^+^ T Cells in Mice

**FIGURE 11:**
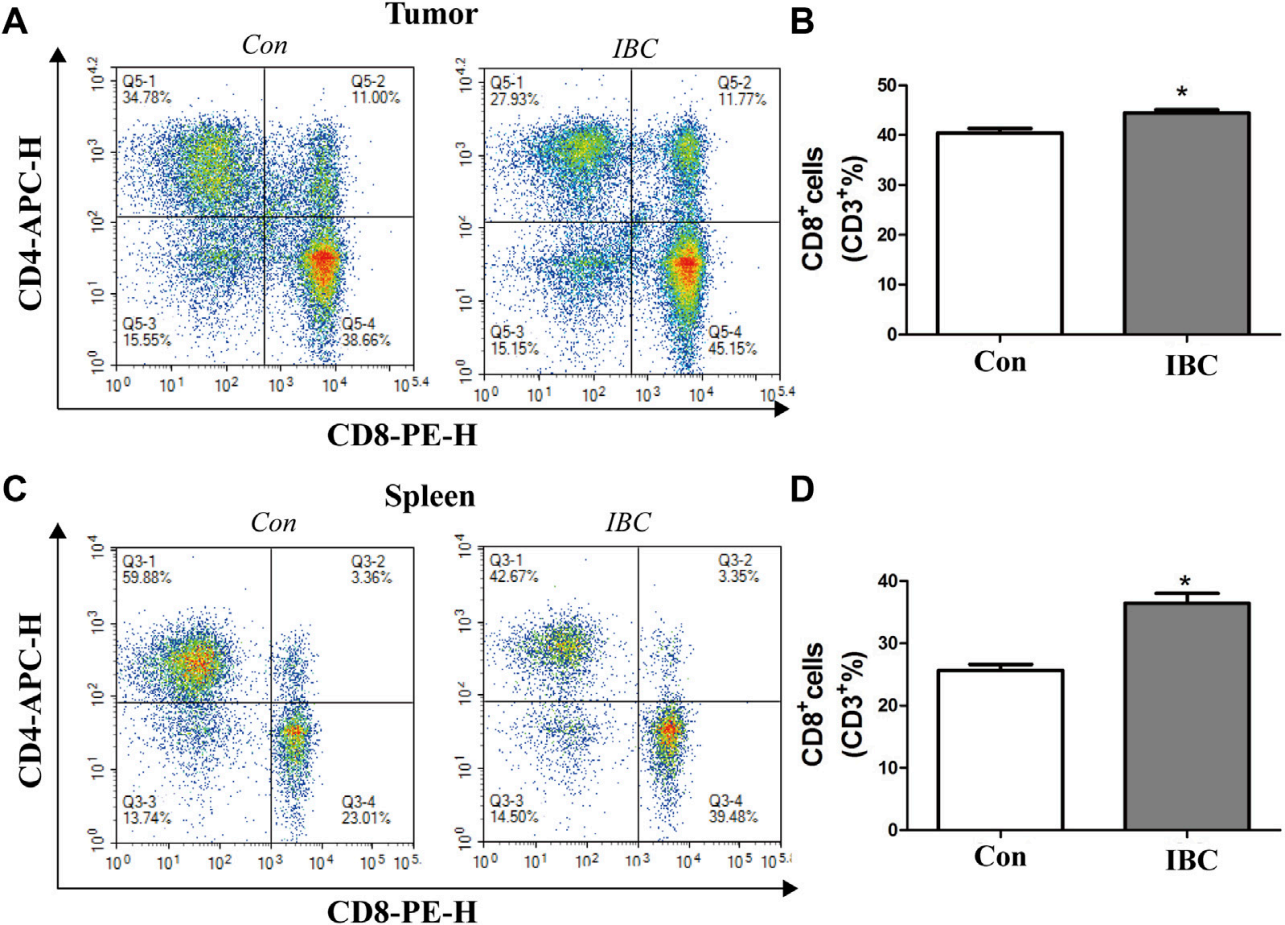
**(A,B)** In tumor tissues, the representative image of the CD8^+^ proportion was reported. The columns represent the mean ± SEM. Student’s *t*-test was applied to analyze the data, *p < 0.05 versus the control. **(C,D)** Proportion of CD8^+^ in the spleen was reported, *p < 0.05 versus the control.

Natural killer (NK) cells, CD4^+^ T cells, and CD8^+^ T cells play essential roles in cancer development. We evaluated their ratio in tumor tissues and spleen by flow cytometry and confirmed that IBC could significantly increase the accumulation of CD8^+^ T cells in tumor tissue and spleen (Figure 11), while IBC did not affect the frequencies of CD4^+^ T cells and NK cells (Supplementary Figure S2).

## Discussion

Partially as a result of the aging population and improvement of treatments in other cancers, pancreatic cancer is increasing per year and is perhaps the second leading cause of cancer death (Neoptolemos et al., 2018). The direct deficiency of the standard treatment is the high rate of primary irresectability and surgical complications, and doctors widely accepted neoadjuvant concepts with antitumor drugs (Heinrich and Lang, 2017). Although widespread efforts have been made to develop antitumor agents for pancreatic cancer, limited efficacy and tolerance have meant that searching for a new natural compound with potent and alternative antitumor activity is imperative (Zeng et al., 2019).

In this research, we established a method to screen for a valuable antitumor agent from TCM through network pharmacology, bioinformatics, and molecular docking. Next, we validated the pharmacologic effects on the animal model. Taking the PCL as an attempt, we collected active components of PCL to select a compound that may mediate resistance to pancreatic cancer growth. Following the network pharmacology and bioinformatics, we achieved 13 compounds and 10 core targets and considered IBC as the candidate. In addition, MAPK1, FYN, and PIK3CA were the important constituents of core targets and were shown to be independent prognostic factors. MAPK1 plays a vital role in the tumor microenvironment of various cancers. A previous study demonstrated that MAPK1 is upregulated and hypersensitive to the recurrence of head and neck squamous cell carcinoma (Watermann et al., 2021). Secreted exosomal CMTM6 of oral squamous cell carcinoma induces M2-like macrophage polarization and promotes malignant progression *via* the MAPK3/MAPK1 signaling pathway (Pang et al., 2021). Among ovarian cancer patients, FYN is one of the indispensable kinase targets of CXC chemokines, which activate the downstream signaling pathways to influence tumor progression (Jin et al., 2021). In addition, the TIMER database suggested that FYN, MAPK, and PIK3CA were positively correlated with immune infiltration of B cells, CD8^+^ T cells, macrophages, neutrophils, and dendritic cells. Based on these data, we proposed that IBC can functionally sculpt the tumor microenvironment by regulating the proportion of immune cells.

During the validation phase, our study showed that IBC could inhibit proliferation of Panc 02, promote cell apoptosis *via* increasing the level of ROS production, and attenuate the weights of orthotopic cancer by inhibiting tumor proliferation and increasing tumor apoptosis directly, which is in line with the previous studies (Jing et al., 2010; Li et al., 2018; Li et al., 2019). Furthermore, we focused on the effects of IBC treatment on the tumor microenvironment of pancreatic cancer. The formation of the tumor microenvironment, induced by interactions between pancreatic epithelial/cancer cells and stromal cells, contributes to desmoplasia and immunosuppression and is always accompanied by poor prognosis (Ren et al., 2018). M2 macrophages and MDSC considerably govern the unfavorable tumor microenvironment and are considered obstacles in immunotherapy (Groth et al., 2019).

M2 macrophages, one of the critical components of the tumor microenvironment, highly accumulate in tumor tissues and participate in cancer progression, simultaneous immunosuppression, angiogenesis, and drug resistance (Qian and Pollard, 2010; Zhao et al., 2020). Our data revealed that both M2 macrophages in tumor tissues and spleen significantly decreased administrated by IBC. MDSC, a heterogeneous group of immune cells, are derived from the bone marrow and transferred to the solid tumor and peripheral lymphoid organs to contribute to the formation of the tumor microenvironment (Kumar et al., 2016). Along with inherent immunosuppressive activity, it can amplify the functions of macrophages and dendritic cells and promote carcinoma progression *via* cell-cell interaction (Ostrand-Rosenberg et al., 2012). In this research, it was found that a reduction of MDSC in tumor tissues, in which IBC could allow for improved tumor progression *via* inhibiting the mRNA expression of ARG1 and TGF-β. However, the proportion of MDSC in the spleen has not changed. In addition to this, CD8^+^ cells and NK cells mediate the antineoplastic effect and play vital roles in the immune response. This research found a reduction of CD8^+^ in tumor and spleen tissues, but the proportion of NK cells did not change. The aforementioned results indicate that the function and fate of MDSC remain different in the tumor and peripheral lymphoid organs of pancreatic cancer, and IBC can orchestrate M2 macrophage, MDSC, and CD8^+^ T cells to serve as an antineoplastic agent.

## Conclusion

In summary, we demonstrated that IBC treatment inhibits Panc 02 cell proliferation, promotes cell apoptosis *via* increasing production of ROS, and activates antitumor immunity. It provides a new approach to elucidate the antitumor activity of IBC and could serve as a reference for treating the tumor microenvironment in pancreatic cancer.

## Data Availability

Publicly available datasets were analyzed in this study. This data can be found at: https://gdc-hub.s3.us-east-1.amazonaws.com/download/TCGA-PAAD.htseq_counts.tsv.gz.
