# Supplementary material for: Isobavachalcone Activates Antitumor Immunity on Orthotopic Pancreatic Cancer Model: A Screening and Validation
Source: Front Pharmacol. 2022 Aug 25;13:919035. doi: 10.3389/fphar.2022.919035 (PMC9452641; doi:10.3389/fphar.2022.919035)
Supplement: Supplementary file 1 [file DataSheet1.zip › Supplementary materials/Supplementary Table 1.docx]

**Supplementary Table 1.**

The Information of 28 Compounds of PCL from TCMID.

| No. | Compounds | SMILE formats | Target Number |
| --- | --- | --- | --- |
| 1 | Angelicin | C1=CC2=C(C=CO2)C3=C1C=CC(=O)O3 | 46 |
| 2 | Bakuchalcone | CC(C)(C1CC2=C(O1)C=CC(=C2O)C(=O)C=CC3=CC=C(C=C3)O)O | 61 |
| 3 | Bakuchiol | CC(=CCCC(C)(C=C)C=CC1=CC=C(C=C1)O)C | 78 |
| 4 | Bavachalcone | CC(=CCC1=CC(=C(C=C1O)O)C(=O)C=CC2=CC=C(C=C2)O)C | 100 |
| 5 | Bavachin | CC(=CCC1=CC2=C(C=C1O)OC(CC2=O)C3=CC=C(C=C3)O)C | 100 |
| 6 | Bavachinin | CC(=CCC1=CC2=C(C=C1OC)OC(CC2=O)C3=CC=C(C=C3)O)C | 100 |
| 7 | Bavacoumestan_a | CC1(C(CC2=CC3=C(C=C2O1)OC4=C3C(=O)OC5=C4C=CC(=C5)O)O)C | 12 |
| 8 | Bavacoumestan_b | CC(C)(C1CC2=CC3=C(C=C2O1)OC4=C3C(=O)OC5=C4C=CC(=C5)O)O | 12 |
| 9 | Corylidin | CC1(C(C(C2=C(O1)C=C3C(=C2)C4=C(C5=C(O4)C=C(C=C5)O)C(=O)O3)O)O)C | 15 |
| 10 | Corylin | CC1(C=CC2=C(O1)C=CC(=C2)C3=COC4=C(C3=O)C=CC(=C4)O)C | 41 |
| 11 | Corylinal | C1=CC(=C(C=C1C2=COC3=C(C2=O)C=CC(=C3)O)C=O)O | 46 |
| 12 | Daucosterol | CCC(CCC(C)C1CCC2C1(CCC3C2CC=C4C3(CCC(C4)OC5C(C(C(C(O5)CO)O)O)O)C)C)C(C)C | 16 |
| 13 | Docosanoic acid | CCCCCCCCCCCCCCCCCCCCCC(=O)O | 29 |
| 14 | Isobavachalcone | CC(=CCC1=C(C=CC(=C1O)C(=O)C=CC2=CC=C(C=C2)O)O)C | 100 |
| 15 | Isobavachin | CC(=CCC1=C(C=CC2=C1OC(CC2=O)C3=CC=C(C=C3)O)O)C | 100 |
| 16 | Isoneobavachalcone | COC1=C(C=C(C(=C1)O)C=O)C(=O)C=CC2=CC=C(C=C2)O | 62 |
| 17 | Isopsoralidin | CC1(CCC2=CC3=C(C=C2O1)OC(=O)C4=C3OC5=C4C=CC(=C5)O)C | 31 |
| 18 | Isposoralen | C1=CC2=C(C=CO2)C3=C1C=CC(=O)O3 | 20 |
| 19 | Neobavachalcone | COC1=CC(=C(C=C1C=O)C(=O)C=CC2=CC=C(C=C2)O)O | 38 |
| 20 | Psoraldehyde | CC=CC=CC=O | 4 |
| 21 | Psoralen | C1=CC(=O)OC2=CC3=C(C=CO3)C=C21 | 20 |
| 22 | Psoralenol | CC1(C(CC2=C(O1)C=CC(=C2)C3=COC4=C(C3=O)C=CC(=C4)O)O)C | 10 |
| 23 | Psoralidin | CC(=CCC1=CC2=C(C=C1O)OC(=O)C3=C2OC4=C3C=CC(=C4)O)C | 27 |
| 24 | Sophoracoumestan | COC1=C(C=CC2=C1OC(=O)C3=C2OC4=CC5=C(C=C43)OCO5)O | 11 |
| 25 | Stearicacid | CCCCCCCCCCCCCCCCCC(=O)O | 64 |
| 26 | Stigmasterol | CCC(C=CC(C)C1CCC2C1(CCC3C2CC=C4C3(CCC(C4)O)C)C)C(C)C | 28 |
| 27 | Triacontane | CCCCCCCCCCCCCCCCCCCCCCCCCCCCCC | 1 |
| 28 | Xanthotoxin | COC1=C2C(=CC3=C1OC=C3)C=CC(=O)O2 | 20 |
