# Supplementary material for: Isobavachalcone Activates Antitumor Immunity on Orthotopic Pancreatic Cancer Model: A Screening and Validation
Source: Front Pharmacol. 2022 Aug 25;13:919035. doi: 10.3389/fphar.2022.919035 (PMC9452641; doi:10.3389/fphar.2022.919035)
Supplement: Supplementary file 1 [file DataSheet1.zip › Supplementary materials/Supplementary Table 2.docx]

**Supplementary Table 2.**

The information of 13 active compounds and 10 core targets of pancreatic cancer.

| Catogory | No. | Name | Degree | Closeness Centrality | Betweenness Centrality | CAS/PubChem CID |
| --- | --- | --- | --- | --- | --- | --- |
| Active  compounds | 1 | Bavachalcone | 101 | 0.3927 | 0.01788 | 28448-85-3 |
|  | 2 | Bavachin | 101 | 0.3927 | 0.01819 | 19879-32-4 |
|  | 3 | Bavachinin | 101 | 0.3927 | 0.01892 | 19879-30-2 |
|  | 4 | Isobavachalcone | 101 | 0.3927 | 0.01381 | 20784-50-3 |
|  | 5 | Isobavachin | 101 | 0.3927 | 0.01848 | 31524-62-6 |
|  | 6 | Bakuchiol | 79 | 0.3799 | 0.01132 | 10309-37-2 |
|  | 7 | Stearic acid | 65 | 0.3721 | 0.00913 | 57-11-4 |
|  | 8 | Isoneobavachalcone | 63 | 0.3710 | 0.00701 | 5318608 |
|  | 9 | Bakuchalcone | 62 | 0.3705 | 0.00802 | 84575-13-3 |
|  | 10 | Backuchiol | 53 | 0.3657 | 0.00446 | 50918968 |
|  | 11 | Angelicin | 47 | 0.3625 | 0.00442 | 523-50-2 |
|  | 12 | Corylinal | 47 | 0.3625 | 0.00369 | 44257227 |
|  | 13 | Corylin | 42 | 0.3620 | 0.00292 | 53947-92-5 |
| Core targets | 1 | PIK3CA | 69 | 0.4351 | 0.09281 | NA |
|  | 2 | SRC | 67 | 0.4368 | 0.06995 | NA |
|  | 3 | MAPK1 | 61 | 0.4415 | 0.10160 | NA |
|  | 4 | AKT1 | 54 | 0.4278 | 0.06802 | NA |
|  | 5 | APP | 53 | 0.3962 | 0.09400 | NA |
|  | 6 | STAT3 | 51 | 0.4323 | 0.09301 | NA |
|  | 7 | FYN | 51 | 0.4019 | 0.02411 | NA |
|  | 8 | LCK | 49 | 0.4019 | 0.01269 | NA |
|  | 9 | LYN | 43 | 0.3981 | 0.01417 | NA |
|  | 10 | HS90AA1 | 38 | 0.4079 | 0.03475 | NA |
