# Supplementary figures and images for: Isobavachalcone Activates Antitumor Immunity on Orthotopic Pancreatic Cancer Model: A Screening and Validation
Source: Front Pharmacol. 2022 Aug 25;13:919035. doi: 10.3389/fphar.2022.919035 (PMC9452641; doi:10.3389/fphar.2022.919035)

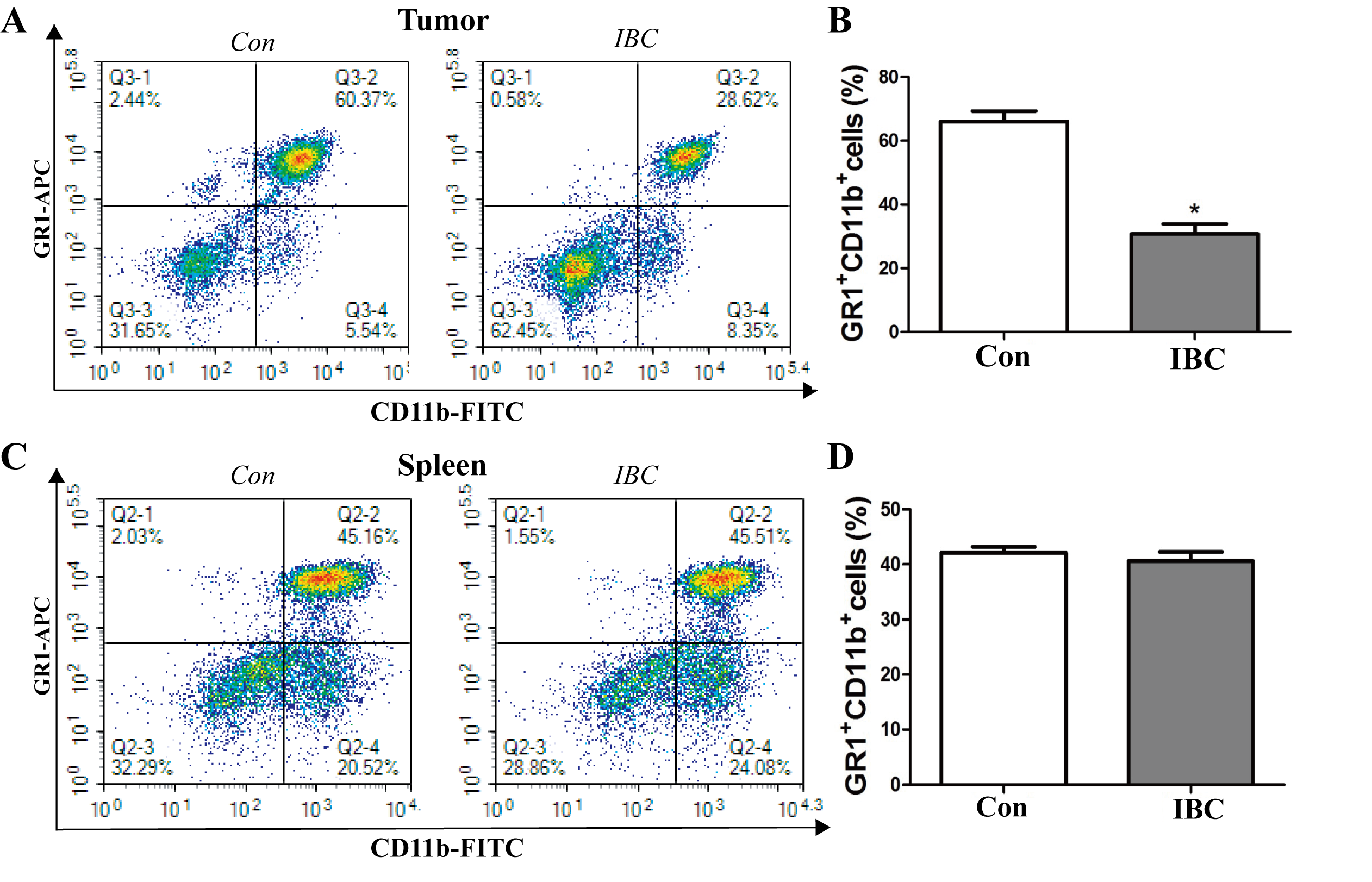

Supplement: Supplementary file 1 [file DataSheet1.zip › Supplementary materials/Figure S1.tif]

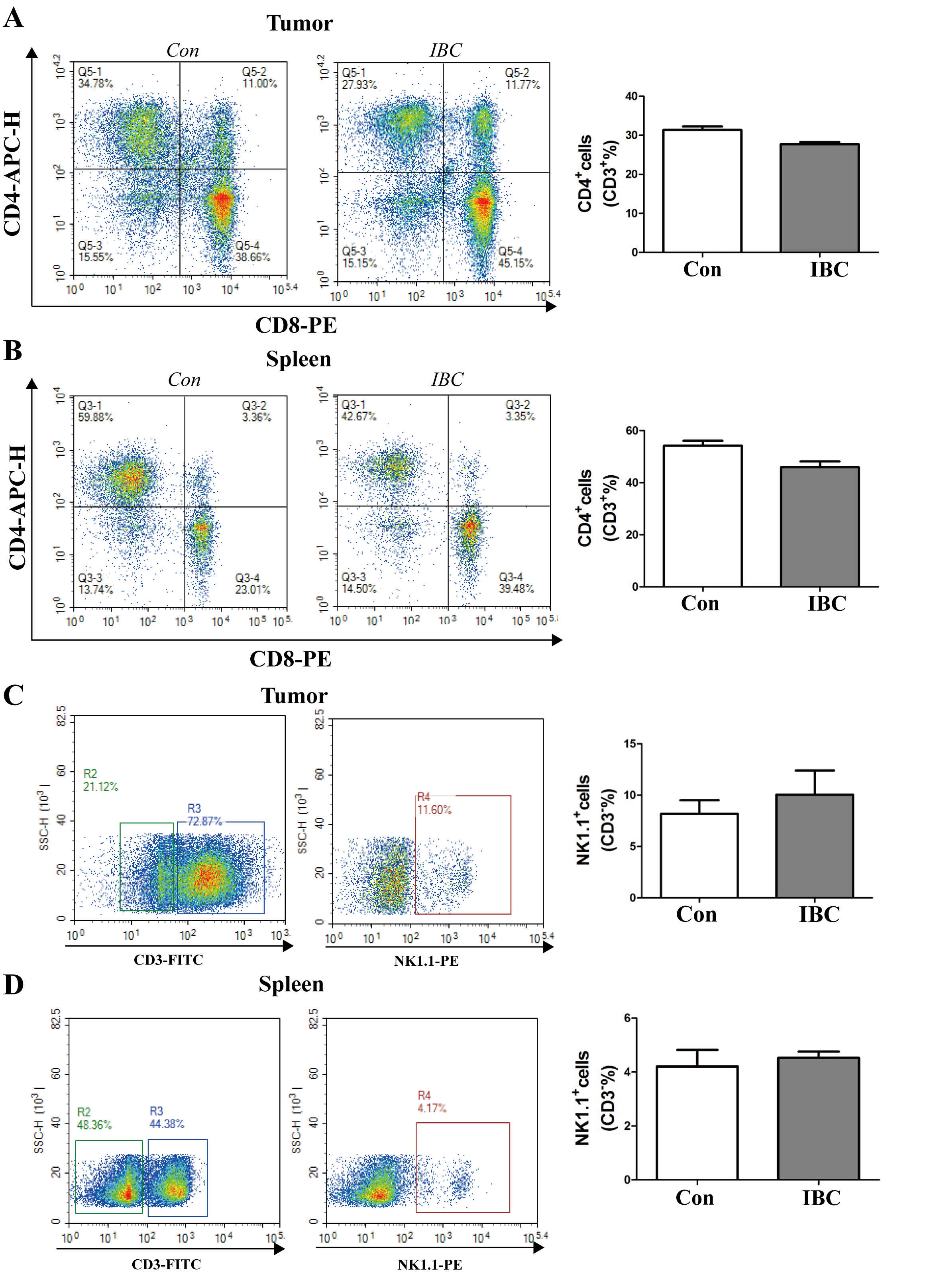

Supplement: Supplementary file 1 [file DataSheet1.zip › Supplementary materials/Figure S2.tif]
